# Ingenious Wife

**Published:** 1872-10

**Authors:** 


AN ingenious wife in Des Moines cured her husband of snoring thus: She has a gutta-percha tube with cup-shaped ends; one she puts over his nose and mouth, and the other over his ear. He consumes his own noise, as a stove does its smoke, and wakes up *instanter.*

